# Alteration of Neutrophil Reactive Oxygen Species Production by Extracts of Devil's Claw (*Harpagophytum*)

**DOI:** 10.1155/2016/3841803

**Published:** 2016-06-27

**Authors:** Mbaki Muzila, Kimmo Rumpunen, Helen Wright, Helen Roberts, Melissa Grant, Hilde Nybom, Jasna Sehic, Anders Ekholm, Cecilia Widén

**Affiliations:** ^1^Department of Plant Breeding, Swedish University of Agricultural Sciences, Balsgård, Fjälkestadsvägen 459, 291 94 Kristianstad, Sweden; ^2^Biological Sciences, University of Botswana, Private Bag UB 00704, Gaborone, Botswana; ^3^School of Dentistry and MRC Centre for Immune Regulation, University of Birmingham, Saint Chads Queensway, Birmingham B4 7ET, UK; ^4^School of Health and Society, University of Kristianstad, 291 88 Kristianstad, Sweden

## Abstract

*Harpagophytum*, Devil's Claw, is a genus of tuberiferous xerophytic plants native to southern Africa. Some of the taxa are appreciated for their medicinal effects and have been traditionally used to relieve symptoms of inflammation. The objectives of this pilot study were to investigate the antioxidant capacity and the content of total phenols, verbascoside, isoverbascoside, and selected iridoids, as well as to investigate the capacity of various* Harpagophytum* taxa in suppressing respiratory burst in terms of reactive oxygen species produced by human neutrophils challenged with phorbol myristate acetate (PMA), opsonised* Staphylococcus aureus,* and* Fusobacterium nucleatum*.* Harpagophytum* plants were classified into different taxa according to morphology, and DNA analysis was used to confirm the classification. A putative new variety of* H. procumbens* showed the highest degree of antioxidative capacity. Using PMA, three* Harpagophytum* taxa showed anti-inflammatory effects with regard to the PBS control. A putative hybrid between* H. procumbens* and* H. zeyheri* in contrast showed proinflammatory effect on the response of neutrophils to* F. nucleatum* in comparison with treatment with vehicle control.* Harpagophytum* taxa were biochemically very variable and the response in suppressing respiratory burst differed. Further studies with larger number of subjects are needed to corroborate anti-inflammatory effects of different taxa of* Harpagophytum*.

## 1. Introduction

Many diseases have an inflammatory pathogenesis and a large number of plant species have for centuries been utilized for their anti-inflammatory and healing effects. The medicinal tuberiferous xerophyte Devil's Claw (*Harpagophytum*) belongs to the family Pedaliaceae and is native to southern Africa [1]. This genus includes two species:* Harpagophytum procumbens* and* H. zeyheri*. There are also a few known subspecies of both species, as well as novel and hybrid taxa with less clear identity. Extracts made from the tubers of* H. procumbens* are known to relieve symptoms of inflammation and pain [2, 3] but there has been some argument about whether medicinal properties of* H. zeyheri* are sufficient for the acceptance of this species for use in anti-inflammatory preparations [4–6]. The pharmacological actions of Devil's Claw root tubers have been attributed to the presence of iridoid glycosides and verbascoside [4, 7, 8]. Studies have demonstrated anti-inflammatory properties such as inhibition of COX-2, inhibition of NF-kB activation, and downregulation of iNOS [9–11]. However, the effects of Devil's Claw have also been associated with the presence of other compounds such as flavonoids [11].

Neutrophil-mediated oxidant injury is a feature of many inflammatory diseases [12, 13]. The initial response to infection is often mediated by neutrophils because of their rapid chemotactic response towards bacteria [14]. Neutrophils inhibit infection activity by ingestion of microorganisms, synthesis of reactive oxygen species (ROS), and release of cytokines. Neutrophils are important in both the innate and the acquired immune responses and microorganisms are recognized through receptor-mediated mechanisms, for example, by the antibody-antigen complex mediated Fc receptor (FcR) and bacterial product mediated Toll-like receptors (TLR) [15, 16]. These receptors represent specific, adaptive immune responses and nonspecific, innate immune responses, respectively, and activate intracellular signal transduction such as protein kinase C, MAPK cascades, and the NADPH-oxidase enzyme complex [17]. The NADPH-oxidase enzyme complex produces ROS at the expense of NADPH. In resting cells from healthy donors little ROS is produced, limiting potential bystander damage to adjacent tissues. Upon stimulation ROS are produced in the local environment where they react rapidly with proximal molecules. In chronic inflammatory disease, such as severe gum disease (periodontitis), both resting and stimulated neutrophils are hyperactive [18]. Therapeutic strategies are continuously sought to decrease these characteristics and prevent excessive collateral tissue damage in infectious situations without preventing the primary infection fighting capacity. Neutrophils may therefore be used as a model to explore mechanisms and possible therapeutic modulation of inflammation.

The specific objective of this paper was (1) to identify and to select genetically diverse* Harpagophytum* plant material that adequately represents the pharmacological capability of the genus and (2) to determine the potential of root tuber ethanol extracts of various taxa to suppress the production of ROS in human neutrophils. We also investigated the antioxidant capacity and the content of ascorbate, verbascoside, isoverbascoside, major iridoids, and total phenols in the extracts.

## 2. Materials and Methods

### 2.1. DNA Studies and Plant Material Selection

**Figure 1:**
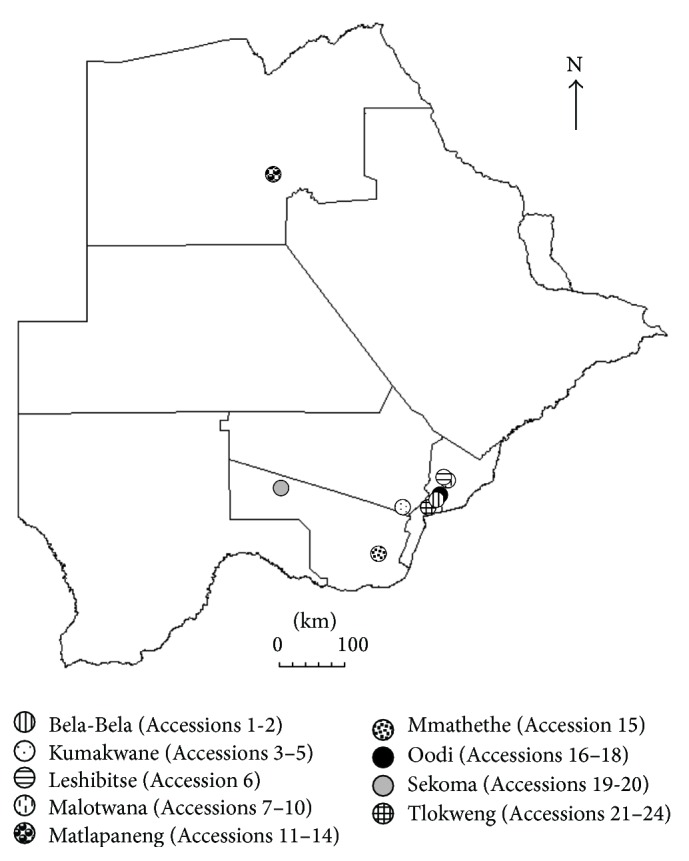
Sampling sites in Botswana for accessions used in the DNA marker, biochemical and neutrophil studies.

Seed capsules (for taxonomic and DNA-based species determination) and secondary root tubers (for analysis of chemical content and* in vitro* studies) of 24* Harpagophytum* accessions were sampled in Botswana (Table 1, Figure 1).

To select genetically diverse samples and to corroborate the taxonomical classification based on morphological characters DNA analyses were performed. One seed capsule was collected from each of the* Harpagophytum* accessions; seeds were germinated and one seedling per accession was used for extraction of DNA. DNA was extracted with the E.Z.N.A.*™* SP DNA mini kit (Omega Bio-Tek, Norcross, GA, USA). DNA quality was determined in 2% agarose gel. The DNA samples were analyzed with 2 inter-simple sequence repeat (ISSR) and 6 random amplified polymorphic DNA (RAPD) primers, using previously described methodology [19]. RAPD and ISSR bands were scored as present or absent, and a total of 107 polymorphic DNA bands were obtained. Based on the DNA studies 6 accessions representing 5 taxa were selected for use in the subsequent neutrophil and biochemical study:* H. procumbens* ssp.* transvaalense *(Accession 17; O1APT), a putative new variety of* H. procumbens* ssp.* transvaalense* (Accession 3; K1APN),* H. zeyheri* ssp.* zeyheri *(Accession 24; T1AZZ), and* H. zeyheri* ssp.* sublobatum* (Accession 14; MP3AZS) and two samples with unclear taxonomic identity but most likely interspecific hybrids between* H. procumbens* and* H. zeyheri* (Accession 16; O1APH and Accession 11; MP1APH) according to morphological characters of the tuber-bearing plants [20].

### 2.2. Root Sample Preparation

Samples of the root tubers were obtained by sectioning the specimen into halves, and the peel and pulp were separated, weighed, and freeze-dried at the Department of Plant Breeding, Balsgård, Swedish University of Agricultural Sciences. Vacuum was applied at <0.2 mbar and the condenser temperature was set at −70°C during the freeze-drying process. The initial temperature of the sample tray was −5°C, and after 16 h a temperature gradient was applied from −35°C to 10°C. The temperature was then maintained at 10°C until the extracts were completely dry. The dry extracts were ground to a fine powder in a laboratory mill (Yellow line, A10, IKA-Werke, Staufen, Germany).

### 2.3. Extract Preparation

Extracts were made fresh before each experiment by adding 1 mL of 50% ethanol containing 0.05 M H_3_PO_4_ to 250 *μ*g (for* in vitro* studies) or 50 mg (for biochemical studies) of finely ground* Harpagophytum* tuber powder. The extracts were kept in an ultrasonic bath for 15 min before centrifugation at 16000 g for 10 min and the supernatant was collected. This resulted in a 100% stock with a concentration of 250 *μ*g/mL. This stock was then diluted (v/v) with phosphate-buffered saline (PBS) to produce final concentrations of 50%, 10%, and 5%.

### 2.4. Determination of Total Phenolics

To measure the total phenolic content according to the Folin-Ciocalteu method [21] the sample was mixed with Folin-Ciocalteu reagent (Merck, Darmstadt, Germany), H_2_O, and 15% Na_2_CO_3_ and the absorbance measured at 765 nm after 1 h incubation at room temperature. Gallic acid was used as a standard and the total content of phenols was expressed as mg gallic acid equivalents (GAE) per g dry weight (dw). For comparison, total phenolic content was analyzed also in commercially obtained, external standards of 8-O-p-coumaroyl-harpagide, harpagoside, and verbascoside.

### 2.5. Determination of Ferric Reducing Ability of Plasma

The ferric reducing ability of plasma (FRAP) of the extracts was measured according to the method developed by Benzie and Strain [22] but modified to fit a 96-well format [23]. The different extracts were diluted 20–100-fold. Ten *μ*L of these extracts was incubated at 37°C and then mixed with 260 *μ*L of ferric-TPTZ reagent (prepared by mixing 300 mM acetate buffer, pH 3.6; 10 mM of 2,4,6-tripyridyl-s-triazine in 40 mM HCl; and 20 mM FeCl_3_ in the ratio of 4 : 1 : 1; the solution was kept at 37°C). The absorbance was measured at 595 nm after 4 min on a plate reader (Sunrise, Tecan Nordic AB, Sweden). Fe^2+^ was used as a standard and L-ascorbic acid was used as a control where one mole of ascorbic acid corresponds approximately to two moles of FRAP (we obtained the value 2.09).

### 2.6. HPLC Analysis of Ascorbate, Verbascoside, Isoverbascoside, and Iridoids

For ascorbate analysis samples were placed in ultrasonic bath for 10 min and centrifuged for 10 min at 2000 g (Beckham, USA). The supernatant was filtered with a syringe particle filter (glass/nylon 0.45 *μ*m, 30 mm Cameo, Sorbent AB, Sweden) directly into an HPLC-vial. The analysis was made on a Shimadzu HPLC system consisting of a communication bus module (SCL 10A-VP) and a pump (LC 10AD) using an ACE (5 *μ*m) for separation. For detection a variable SPD-10A UV-VIS detector set to 254 nm was used. The mobile phase consisted of a 50 mM sodium phosphate (Fluka, Switzerland) buffer set to pH 2.8. The flow rate was 1 mL min^−1^ and 20 *μ*L sample was injected into the HPLC system. The peak was identified by retention time of an ascorbate standard (Sigma-Aldrich, USA). Quantification was carried by peak area.

Contents of verbascoside, isoverbascoside, and the iridoids acetylacteoside, 8-*O*-p-coumaroyl-harpagide, harpagoside, and pagoside were analyzed in* Harpagophytum* extracts on a Shimadzu HPLC system equipped with a diode-array detector according to a method slightly modified from Karioti et al. [24]. The eluent consisted of solvent A (H_2_O at pH 3.2 by formic acid) and solvent B (acetonitrile). The binary gradient was as follows: 95% A (0–5 min), 85% A (5–8 min), 76% A (8–15 min), 75% A (15–19 min), 73% A (19–24 min), 71% A (24–29 min), and 50% A (29 min). A Phenomenex Synergi 4l Hydro-RP 80A column (250 × 4.6 mm) and a guard C18 precolumn were used. Evaluation of data was carried out with Shimadzu Class-VP software (version 6.13 SP2). Retention times and spectral data were obtained and compared with those of the external standards verbascoside, 8-O-p-coumaroyl-harpagide, and harpagoside. The peaks of acetylacteoside, pagoside, and isoverbascoside were verified by HPLC MS. Acetylacteoside and pagoside were quantified against harpagoside, and isoverbascoside was quantified against verbascoside. Detection was carried out at 280 nm with a total run time of 35 minutes. Example chromatograms of accessions are provided as Supplementary Material (Supplementary File, Figure S1, available online at http://dx.doi.org/10.1155/2016/3841803).

### 2.7. Collection of Venous Blood and Preparation of Neutrophils

West Midlands Research Ethics Committee in Birmingham, UK, in compliance with the Declaration of Helsinki, approved the study (Institutional Review Board approval number 10/H1208/48). The study was based on periodontally and systemically healthy individual volunteers (*n* = 10; mean age: 29 years, range 21–61 years, all female) who consented to participate in the study. Exclusion criteria included pregnancy, use of nonsteroidal anti-inflammatory drugs, mouth-washes, antimicrobial drugs, or vitamin supplements within the past three months.

Venous blood was drawn from each participant. A discontinuous Percoll gradient (*δ* = 1.079 : 1.098) was used to isolate neutrophils, followed by erythrocyte lysis using a solution containing 0.83% NH_4_Cl, 1% KHCO_3_, 0.04% EDTA, and 0.25% bovine serum albumin (BSA) for 20 minutes. Isolated cells were then washed and resuspended at 1 × 10^6^ cells/mL in solution grade phosphate-buffered saline (GPBS) containing 1 mM glucose and cations (1 mM MgCl_2_ and 1.5 mM CaCl_2_). Trypan blue was used to determine cell viability and only samples with more than 98% viability were retained for further analysis.

### 2.8. Cell Viability Assay

Cell viability was measured using the CellTiter-Glo*™* Reagent (Promega). The luciferase present in the reagent uses luciferin, oxygen, and ATP as substrates in a reaction that produces oxyluciferin and releases energy in the form of light. Because the luciferase reaction requires ATP, conditions have been created such that the amount of light produced is proportional to the amount of ATP present, reflecting the number of viable cells [25, 26]. 100 *μ*L of neutrophils in GPBS (1 × 10^5^ cells/mL) and 10 *μ*L of PBS, the vehicle control, or the* Harpagophytum* extracts (5%) were added to preblocked (PBS containing 1% BSA overnight, 4°C) white microwells (Microlite 2, VWR, UK). The plate was placed in the microplate reader and incubated at 30°C for 30 min before the addition of 100 *μ*L of CellTiter-Glo® to each well. The contents of the wells were mixed and returned to the plate reader before reading the luminescence levels at 10 min.

### 2.9. Detection of Reactive Oxygen Species (ROS) by Chemiluminescence Assay

Chemiluminescence assays were performed using luminol to detect total oxygen radical (HOCl and H_2_O_2_) generation (intra- and extracellular) as described in Matthews et al. [27]. All assays were performed at 37°C using a Berthold microplate luminometer (LB96v). Supplemented PBS (35 *μ*L, PBS supplemented with glucose, Ca^+^, and Mg^+^) and luminol (30 *μ*L, 3 mM) were added to preblocked (PBS containing 1% BSA overnight, 4°C) white microwells (Microlite 2, VWR, UK). The plate was then placed into the microplate reader and 100 *μ*L of the isolated neutrophils (1 × 10^5^) was added to each well. Cells were allowed to settle for 30 min at 37°C prior to priming by addition of the extracts (only 5%) or controls for 30 min at 37°C. Neutrophils were then stimulated with either PMA (Phorbol 12-myristate 13-acetate, 25 nM),* Fusobacterium nucleatum* (MOI 1 : 100), opsonised* S. aureus* (MOI 1 : 300), or PBS (control).* S. aureus* (NCTC 6571) had been grown aerobically on mannitol salt agar and inoculated into tryptone soy broth; opsonised* S. aureus* was prepared according to Bergström and Åsman [28] and stored as cell suspension of 1.2 × 10^9^ cells/mL at −80°C. The anaerobic bacteria* F. nucleatum* (Fn; ATCC 10953) were grown at 37°C according to Roberts et al. [29]. The bacteria were washed three times in sterile PBS, heat-treated at 100°C for 10 min, and then diluted to a suspension of 5 × 10^9^ cells/mL and stored at −80°C. PMA was resuspended into DMSO and diluted in PBS. For each subject, all samples were analyzed in triplicate and light emission in relative light units (RLUs) was recorded throughout the experiment.

### 2.10. Statistical Analysis

Based on the total of 107 polymorphic RAPD and ISSR bands, a matrix of standardized covariates was calculated for the 24 samples (6 samples investigated in this study and 18 control samples for taxonomic classification) and used as input variable in a principal component analysis (PCA) conducted with GenAlEx.

Associations between the content of chemical compounds in the six chemically analyzed samples were similarly investigated with PCA. Based on the scree-plot, three PCA components were selected for illustration of between-sample similarity. This analysis was performed using the Minitab 16 software (Minitab, State College, PA, USA).

Descriptive statistics for mean values and standard deviations were calculated on data for the enhanced chemiluminescence assays, and mean values, standardized to the total relative light units (RLUs) for each test subject, respectively, were compared using the Wilcoxon signed-rank test. SPSS 20 for Windows was used for these calculations.

## 3. Results and Discussion

### 3.1. Accession Identification, Diversity, and Selection

**Figure 2:**
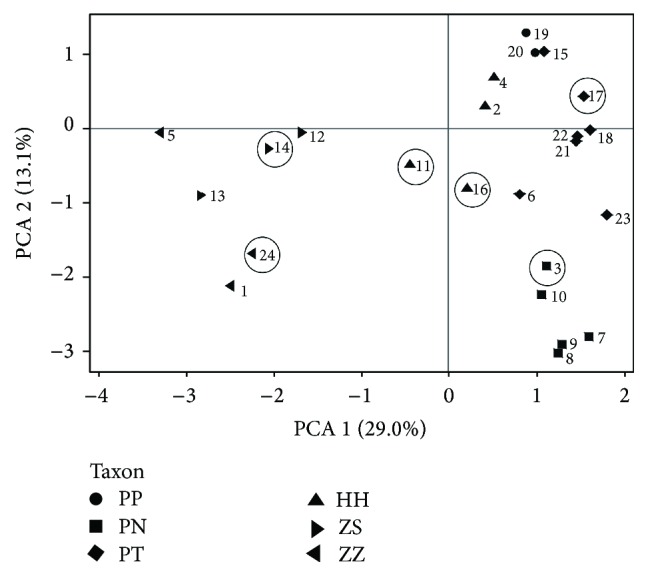
Principal component analysis showing ISSR and RAPD marker-based similarity between 24* Harpagophytum* accessions used in the DNA and neutrophil study (the 6 accessions within circles). Samples are plotted on the first two components, explaining 29 and 13% of the total variation, respectively. The numbers indicate accession codes, whereas the symbols for the taxa are abbreviated as follows: PP:* H. procumbens* ssp.* procumbens*; PT:* H. procumbens* ssp.* transvaalense*; PN: putative new variety of* H. procumbens* ssp.* transvaalense*; ZS:* H. zeyheri* ssp.* sublobatum*; ZZ:* H. zeyheri* ssp.* zeyheri*; HH: putative interspecific hybrids (between* H. procumbens* and* H. zeyheri*).

The first step in this investigation was to ascertain the taxonomic identity of the selected plant material by DNA analysis of seedlings from the field-collected seed capsules. The clustering of the 24 studied accessions in a DNA marker-based PCA (Figure 2) in general supported the morphology-based species classification. Material from six of these accessions (Table 2) was selected to determine the ability to suppress respiratory burst in human neutrophils and to investigate the antioxidant capacity and phenol content. The selected samples of* H. procumbens* ssp.* transvaalense* (Accession 17; O1APT) and of a putative new variety of* H. procumbens* ssp.* transvaalense* (Accession 3; K1APN) grouped with other* H. procumbens* accessions. Similarly, the two samples of* H. zeyheri* ssp.* zeyheri* (Accession 24; T1AZZ) and* H. zeyheri* ssp.* sublobatum* (Accession 14; MP3AZS), respectively, grouped with other accessions of* H. zeyheri*. One of the two samples of putative interspecific hybrids (Accession 11; MP1APH) took an intermediary position while the other sample (Accession 16; O1APH) showed somewhat stronger affinity to the* H. zeyheri* accessions.

### 3.2. Content of Total Phenols

Using the Folin-Ciocalteu assay the highest content of total phenols was found in the putative new variety of* H. procumbens* (Accession 3, 28.9 mg GAE/g dw) and in* H. zeyheri* ssp.* sublobatum* (Accession 14, 25.1 mg GAE/g dw) whereas considerably lower content was revealed for the other samples investigated (Table 2). The external standards of harpagide, harpagoside, and verbascoside were also subjected to this analysis. Low phenolic content was identified for the iridoids and high phenolic content for the verbascoside standard (Table 2).

### 3.3. Antioxidant Capacity of* Harpagophytum* Extracts: Ferric Reducing Activity of Plasma (FRAP)

Using the chemical FRAP assay as a measure of antioxidant capacity, Accession 3 had the highest value (596.7 *μ*mol Fe^2+^/g dw), followed by Accession 14 (444.3 *μ*mol Fe^2+^/g dw), and more than double that of Accession 11 (204.8 *μ*mol Fe^2+^/g dw) (Table 2).

The standards harpagide, harpagoside, and verbascoside were also analyzed and verbascoside was the most potent (5854.2 *μ*mol Fe^2+^/g dw) with approximately 10 × the capacity of harpagoside and 40 × the capacity of harpagide (Table 2).

Thus, there was a very large difference among samples in antioxidant capacity and also the antioxidant capacity of the different standards varied largely.

### 3.4. Content of Verbascoside, Isoverbascoside, Iridoids, and Ascorbate

The content of verbascoside was highest in Accession 14 (mean 4.2 mg/g dw) followed by Accession 3 (mean 3.0 mg/g dw) (Table 3). The content of isoverbascoside was highest in Accession 3 (mean 21.8 mg/g dw). Significant amounts of harpagoside and 8-*O*-p-coumaroyl harpagide were identified in Accession 11 as well as in Accession 14 (Table 3). The extracts from Accessions 16, 17, and 24 contained low levels of verbascoside, isoverbascoside, and iridoid compounds. HPLC analysis revealed that there was no ascorbate in the samples (Supplementary File, Figure S2).

Thus, the phytochemical analysis revealed substantial differences in chemical composition between the analyzed taxa but whether these differences are taxon-specific must be ascertained in future studies using a much larger material with several samples of each taxon.

### 3.5. Cell Viability Assay

There were no significant differences among the effects of different extracts on neutrophil cell viability, and none of the extracts showed lethal/suppressive effects on neutrophils (Supplementary File, Figure S3).

### 3.6. Effect on Neutrophil Reactive Oxygen Species (ROS) Production

To assess the effects of the extracts on neutrophil ROS production, neutrophils from healthy volunteers were preincubated with extracts (5% solutions, including 2.5% ethanol) or controls (PBS or vehicle control 2.5% ethanol) for 30 min prior to stimulation.

**Figure 3:**
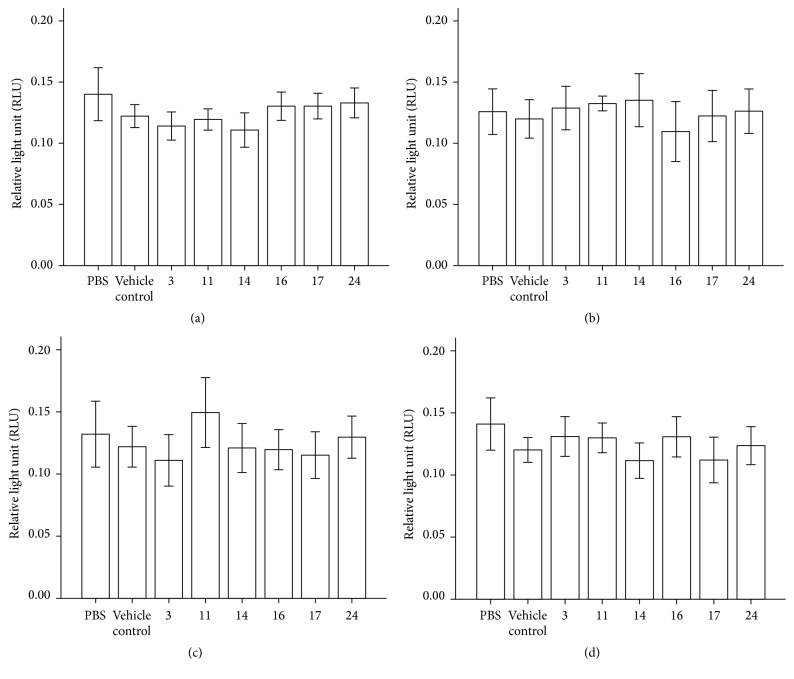
Luminol-detected total ROS production by neutrophils stimulated with (a) PBS (no stimulus), (b) opsonised* Staphylococcus aureus*, (c)* Fusobacterium nucleatum*, and (d) PMA (25 nM) in the presence of PBS, ethanol, and Accessions 3, 11, 14, 16, 17, and 24 (*n* = 10). Error bars: 95% CI. All sample results were compared by Wilcoxon two related samples tests.

For unstimulated cells (PBS, Figure 3(a)), there were significant differences in response among some of the samples. Accession 3 responded significantly stronger (lower RLUs recorded) than Accessions 16 (*p* = 0.028) and 24 (*p* = 0.037), and Accession 14 was significantly stronger than Accessions 16 (*p* = 0.022), 17 (*p* = 0.028), and 24 (*p* = 0.047) in protecting the cells. However, none of the samples were significantly different from the PBS or vehicle ethanol control.

For stimulated cells,* Staphylococcus aureus* treatment (Figure 3(b)), there was a significant proinflammatory effect of Accession 11 in comparison to Accession 16 (*p* = 0.028). A significant proinflammatory response was also detected for Accession 14 with regard to Accession 17 (*p* = 0.017). However, none of the samples were significantly different from the PBS or vehicle ethanol control.

With the Toll-like receptor ligand* F. nucleatum* treatment (Figure 3(c)), a significant proinflammatory response was detected for Accession 11 in comparison with the vehicle control (*p* = 0.037) and also in comparison with Accession 17 (*p* = 0.017).

With PMA treatment (Figure 3(d)), Accessions 14, 16, and 17 showed significant anti-inflammatory activity in comparison to the PBS control (*p* = 0.038, *p* = 0.028, and *p* = 0.049) but no significant difference was detected in comparison with the vehicle control. There was also a significant difference in anti-inflammatory effects among samples. Accessions 14 (*p* = 0.029) and 17 (*p* = 0.037) had significantly higher activity than Accession 3, and Accessions 14 (*p* = 0.015) and 17 (*p* = 0.031) had significantly higher activity than Accession 11.

**Figure 4:**
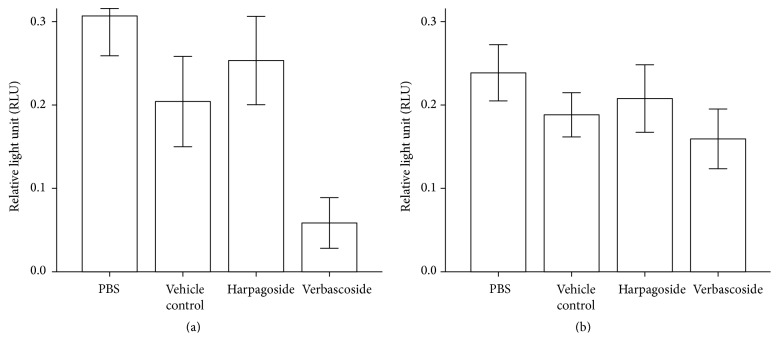
Luminol-detected total ROS production by neutrophils stimulated with (a) PBS or (b) PMA (25 nM) in the presence of PBS, ethanol, harpagoside (0.5 *μ*g/mL), and verbascoside (0.5 *μ*g/mL) (*n* = 5). Error bars: 95% CI. All sample results were compared by Wilcoxon two related samples tests.

Neutrophils were also preincubated with standard solutions (0.5 *μ*g/mL verbascoside and 0.5 *μ*g/mL harpagoside). For unstimulated cells (PBS, Figure 4(a)), significant effects against ROS production were observed for harpagoside and verbascoside in comparison to PBS control (*p* = 0.043 and *p* = 0.043, resp.). Verbascoside was also significantly different to the vehicle control (*p* = 0.043) and to harpagoside (*p* = 0.043). For stimulated cells,* Staphylococcus aureus* and* F. nucleatum* treatments only identified a significant difference between PBS and vehicle control (*p* = 0.043). PMA treatment (Figure 4(b)) revealed a significant difference between PBS and vehicle control (*p* = 0.043) and also between verbascoside and PBS control and harpagoside (*p* = 0.043 and *p* = 0.043, resp.).

Accessions 3 and 14 contained highest levels of verbascoside and isoverbascoside. Accession 11 contained the highest levels of harpagoside, pagoside, and 8-*O*-p-coumaroyl-harpagide. Ouitas and Heard [30] have previously examined a number of* H. procumbens* extracts for anti-inflammatory activity and, in broad agreement with our study, have shown some proinflammatory responses. The reported anti-inflammatory effect by Qi et al. [31] was not replicated in our report, which may be due to cell specific responses and/or the differing properties of the various compounds available in the extracts. In a previous study by Matthews et al. [18] it was demonstrated that ascorbate may significantly decrease the amount of ROS produced by healthy neutrophils stimulated by PMA; however ascorbate was not detected in our samples. Ethanol is known to decrease ROS production by neutrophils [32] and can increase superoxide production during PMA treatment of neutrophils [33]. In our studies the PMA-mediated respiratory burst was clearly affected by ethanol as shown by the vehicle control.

Bivariate Pearson rank correlation analysis identified significant correlations between total phenols and FRAP (*R* = 0.991, *p* = 0.000) as well as between total phenols, FRAP, and PBS control (luminol-detected total ROS production) with verbascoside (*R* = 0.934, *p* = 0.006; *R* = 0.903, *p* = 0.014; *R* = −0.931, *p* = 0.007) and with isoverbascoside (*R* = 0.985, *p* = 0.000; *R* = 0.999, *p* = 0.000; *R* = −0.846, *p* = 0.034). We also noted significant correlation between total phenols and FRAP with PBS control (luminol-detected total ROS production) (*R* = −0.888, *p* = 0.018; *R* = −0.847, *p* = 0.033). The high positive correlations between the content of total phenols and verbascoside as well as with isoverbascoside and the high negative correlation between total phenols and FRAP as well as with the PBS control show that verbascoside really is an important antioxidant phenol in* Harpagophytum* and verifies the antioxidant activity of samples as revealed in the unstimulated cell PBS control treatment.

The present pilot study revealed a tentative anti-inflammatory effect of three* Harpagophytum* taxa using a modest number of subjects. Future verifying studies using a larger number of subjects should take into account gender and age of volunteers, as well as the impact of single purified constituents of the extracts on respiratory burst process* in vitro*.

## 4. Conclusions

The accessions of different* Harpagophytum* taxa were shown to be biochemically very variable with regard to content of verbascoside, isoverbascoside, and analyzed iridoids, and some accessions had very high antioxidant capacity. However, we were unable to corroborate a general anti-inflammatory effect for* H. procumbens* with the neutrophil model although evidence of anti-inflammatory activity was noticed for three taxa. In addition, the proinflammatory effect noticed for one taxon needs further verification.

## Supplementary Material

The Supplementary Material consists of results from the cell viability assay and the HPLC profile (ascorbate, verbascoside, isoverbascoside and selected iridoids) of the Accessions used in the neutrophil and biochemical study.

## Figures and Tables

**Table 1:** Accessions of *Harpagophytum* subjected to DNA analysis.

Accession	Taxon	Location
(1) B1AZZ	*H. zeyheri *ssp. *zeyheri*	Bela-Bela (24°30.7′S, 26°2.27′E) introgression zone
(2) B1APH	Putative hybrid (between *H. procumbens* and *H. zeyheri*)	Bela-Bela (24°30.7′S, 26°2.27′E) introgression zone
(3) K1APN^*∗*^	Putative new variety of *H. procumbens *ssp. *transvaalense*	Kumakwane (24°37.9′S, 25°41.1′E) introgression zone
(4) K1APH	Putative hybrid (between *H. procumbens* and *H. zeyheri*)	Kumakwane (24°37.9′S, 25°41.1′E) introgression zone
(5) K1AZZ	*H. zeyheri *ssp. *zeyheri*	Kumakwane (24°37.9′S, 25°41.1′E) introgression zone
(6) L1APT	*H. procumbens *ssp. *transvaalense*	Leshibitse (24°16.9′S, 26°9.41′E) introgression zone
(7) ML1APN	Putative new variety of *H. procumbens *ssp. *transvaalense*	Malotwana (24°17.5′S, 26°09.27′E) introgression zone
(8) ML1BPN	Putative new variety of *H. procumbens *ssp. *transvaalense*	Malotwana (24°17.5′S, 26°09.27′E) introgression zone
(9) ML2APN	Putative new variety of *H. procumbens *ssp. *transvaalense*	Malotwana (24°17.5′S, 26°09.27′E) introgression zone
(10) ML3APN	Putative new variety of *H. procumbens *ssp. *transvaalense*	Malotwana (24°17.5′S, 26°09.27′E) introgression zone
(11) MP1APH^*∗*^	Putative hybrid (between *H. procumbens* and *H. zeyheri*)	Matlapaneng (19°55.4′S, 23°32.9′E) *H. zeyheri *ssp. *sublobatum* allopatric zone
(12) MP1AZS	*H. zeyheri *ssp. *sublobatum*	Matlapaneng (19°55.4′S, 23°32.9′E) *H. zeyheri *ssp. *sublobatum* allopatric zone
(13) MP2AZS	*H. zeyheri *ssp. *sublobatum*	Matlapaneng (19°55.4′S, 23°32.9′E) *H. zeyheri *ssp. *sublobatum* allopatric zone
(14) MP3AZS^*∗*^	*H. zeyheri *ssp. *sublobatum*	Matlapaneng (19°55.4′S, 23°32.9′E) *H. zeyheri *ssp. *sublobatum* allopatric zone
(15) MT1APT	*H. procumbens *ssp. *transvaalense*	Mmathethe *H. procumbens* ssp. *transvaalense* allopatric zone
(16) O1APH^*∗*^	Putative hybrid (between *H. procumbens* and *H. zeyheri*)	Oodi (24°28.1′S, 26°2.83′E) introgression zone
(17) O1APT^*∗*^	*H. procumbens *ssp. *transvaalense*	Oodi (24°28.1′S, 26°2.83′E) introgression zone
(18) O2APT	*H. procumbens *ssp. *transvaalense*	Oodi (24°28.1′S, 26°2.83′E) introgression zone
(19) S1APP	*H. procumbens *ssp*. procumbens*	Sekoma (24°24.8′S, 23°47.8′E) *H. procumbens *ssp. *procumbens* allopatric zone
(20) S2APP	*H. procumbens *ssp*. procumbens*	Sekoma (24°24.8′S, 23°47.8′E) *H. procumbens *ssp. *procumbens* allopatric zone
(21) T1APT	*H. procumbens *ssp. *transvaalense*	Tlokweng (24°37.8′S, 25°59.1′E) introgression zone
(22) T1BPT	*H. procumbens *ssp. *transvaalense*	Tlokweng (24°37.8′S, 25°59.1′E) introgression zone
(23) T2APT	*H. procumbens *ssp. *transvaalense*	Tlokweng (24°37.8′S, 25°59.1′E) introgression zone
(24) T1AZZ^*∗*^	*H. zeyheri *ssp. *zeyheri*	Tlokweng (24°37.8′S, 25°59.1′E) introgression zone

^*∗*^Accessions used in the neutrophil and biochemical study.

**Table 2:** Content of total phenols (TP) and antioxidant capacity (FRAP) of *Harpagophytum* extracts, presented as mean with standard deviation per dry weight (dw) of the plant material (GAE: gallic acid equivalents).

Accession	Total phenols (mg GAE/g dw)		FRAP (*µ*mol Fe^2+^/g dw)	
	Mean	SD	Mean	SD
3	28.9	0.4	596.7	25.2
11	13.5	0.6	204.8	0.1
14	25.1	1.3	444.3	13.0
16	5.1	0.3	80.8	13.2
17	2.1	0.4	33.8	0.2
24	3.5	0.5	54.5	5.6

Standard: harpagide	16.7	1.1	149.2	0.0
Standard: harpagoside	0.0	0.0	612.3	3.2
Standard: verbascoside	359.9	9.8	5854.2	52.7

**Table 3:** Composition of verbascosides and iridoids in each *Harpagophytum* tuber extract presented as mean value with standard deviation (SD) as mg per g dry weight of the plant material.

Accession	Verbascoside		Isoverbascoside		Acetylacteoside		Pagoside		Harpagoside		8-*O*-p-Coumaroyl-harpagide	
	Mean	SD	Mean	SD	Mean	SD	Mean	SD	Mean	SD	Mean	SD
3	3.04	0.11	21.75	1.16	4.49	0.16	0.11	0.01	4.09	0.34	0.04	0.00
11	1.28	0.11	6.02	0.50	3.30	0.45	0.68	0.08	9.08	0.97	4.04	0.41
14	4.22	0.33	15.62	1.32	1.29	0.15	0.48	0.05	7.07	0.60	3.29	0.35
16	0.64	0.09	2.47	0.27	0.84	0.03	0.17	0.01	0.96	0.15	0.07	0.01
17	0.33	0.05	0.62	0.08	0.23	0.03	0.02	0.00	1.71	0.24	0.09	0.01
24	0.39	0.08	0.64	0.14	0.41	0.11	0.00	0.00	0.16	0.01	0.01	0.00
