# Supplementary material for: Alteration of Neutrophil Reactive Oxygen Species Production by Extracts of Devil's Claw (Harpagophytum)
Source: Oxid Med Cell Longev. 2016 Jun 27;2016:3841803. doi: 10.1155/2016/3841803 (PMC4939354; doi:10.1155/2016/3841803)
Supplement: Supplementary file 1 — The Supplementary Material consists of results from the cell viability assay and the HPLC profile (ascorbate, verbascoside, isoverbascoside and selected iridoids) of the Accessions used in the neutrophil and biochemical study. [file 3841803.f1.pdf]

Supplementary file

Analysis of ascorbate content

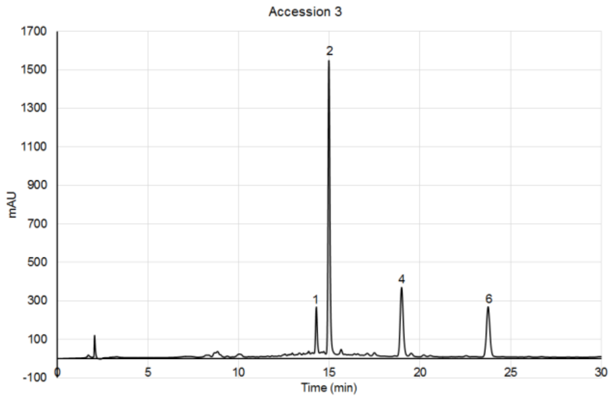

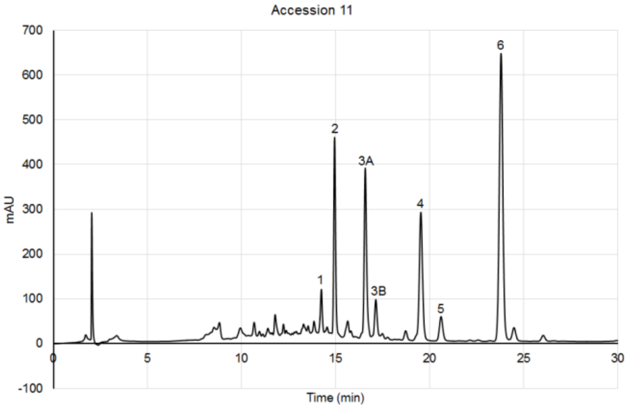

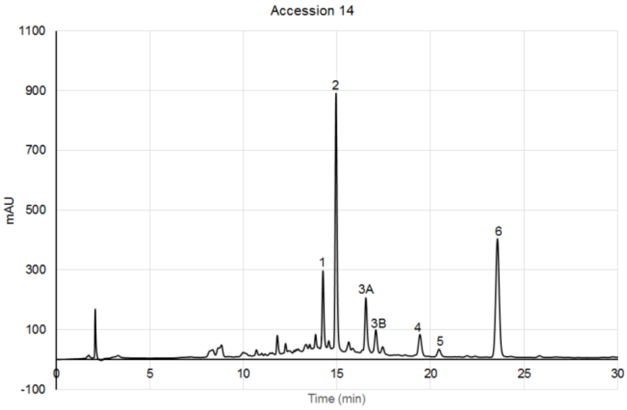

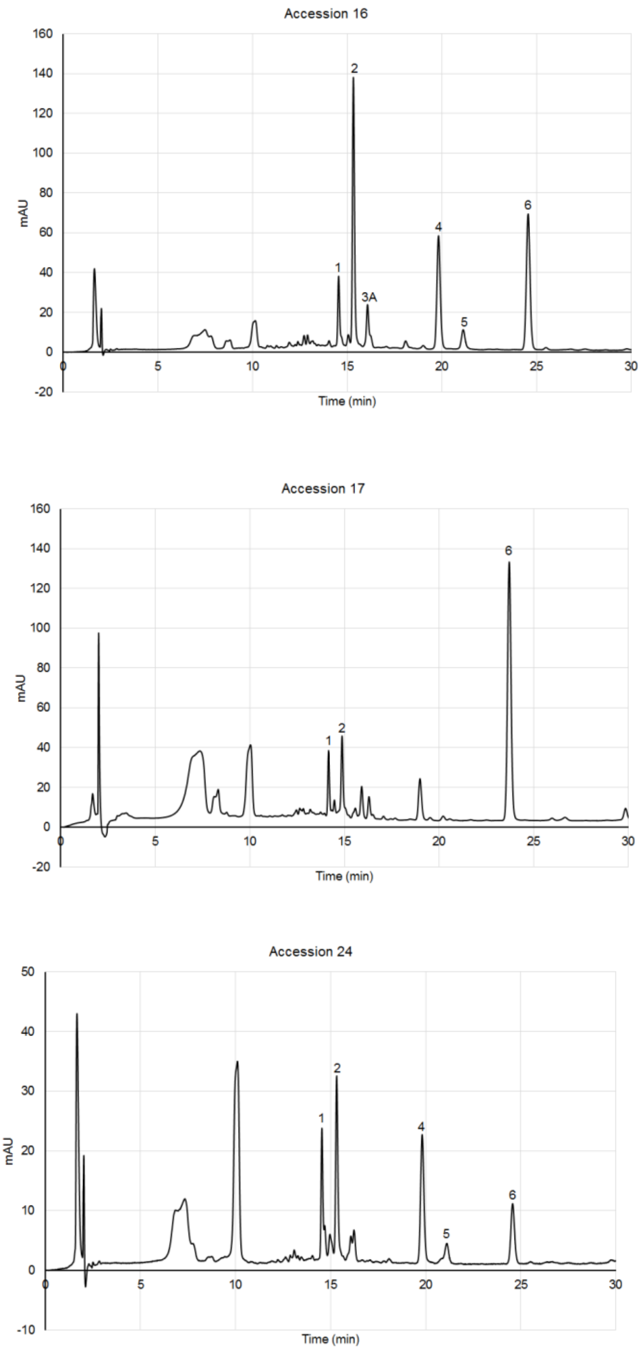

Supplementary Figure S1. Representative HPLC-chromatograms for extracts of the six accessions (Accession 3, 11, 14, 16, 17 and 24) selected for biochemical and *in vitro* analyses. Numbers above the peaks in the chromatogram refer to quantified compounds: 1=verbascoside, 2=isoverbascoside, 3A and 3B=8-*O*-p-coumaroyl-harpagide, 4=acetylacteoside, 5=pagoside and 6=harpagoside.

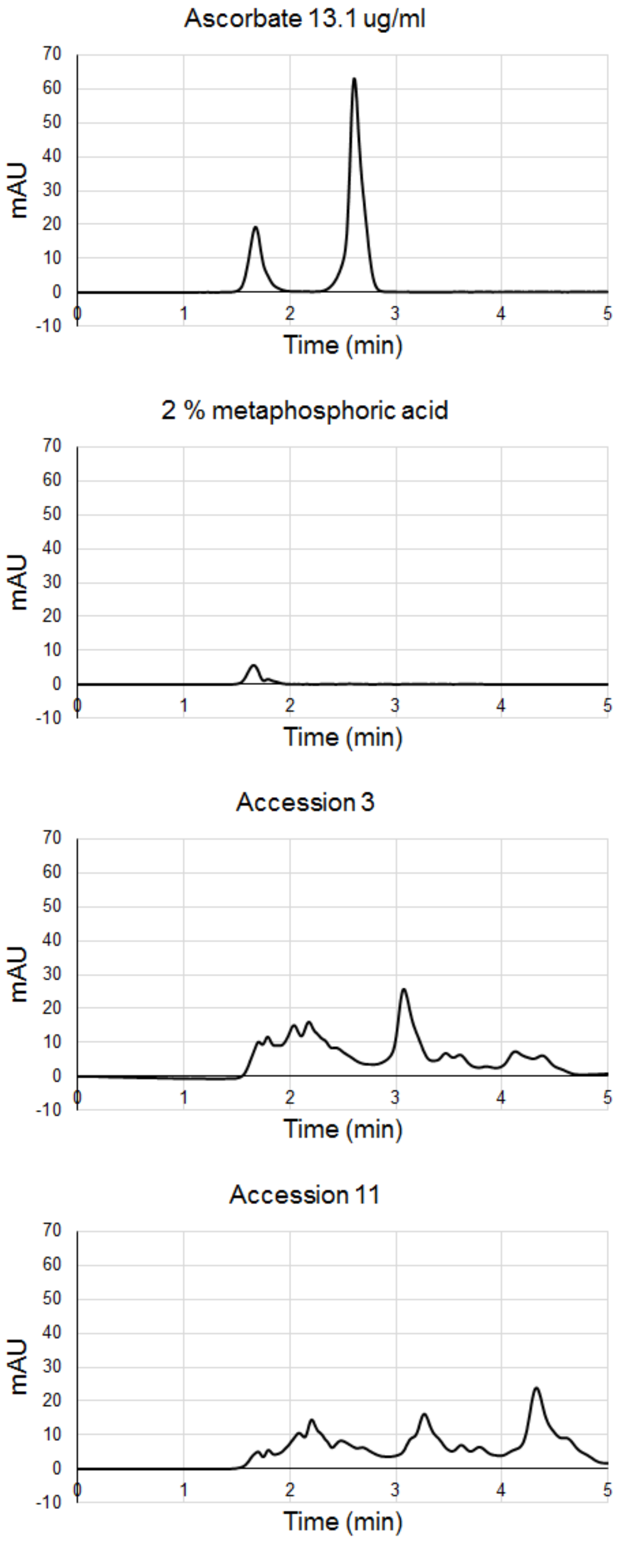
Ascorbate 13.1 ug/ml
2 % metaphosphoric acid
Accession 3
Accession 11
mAU
Time (min)
70
60
50
40
30
20
10
0
-10
0
1
2
3
4
5

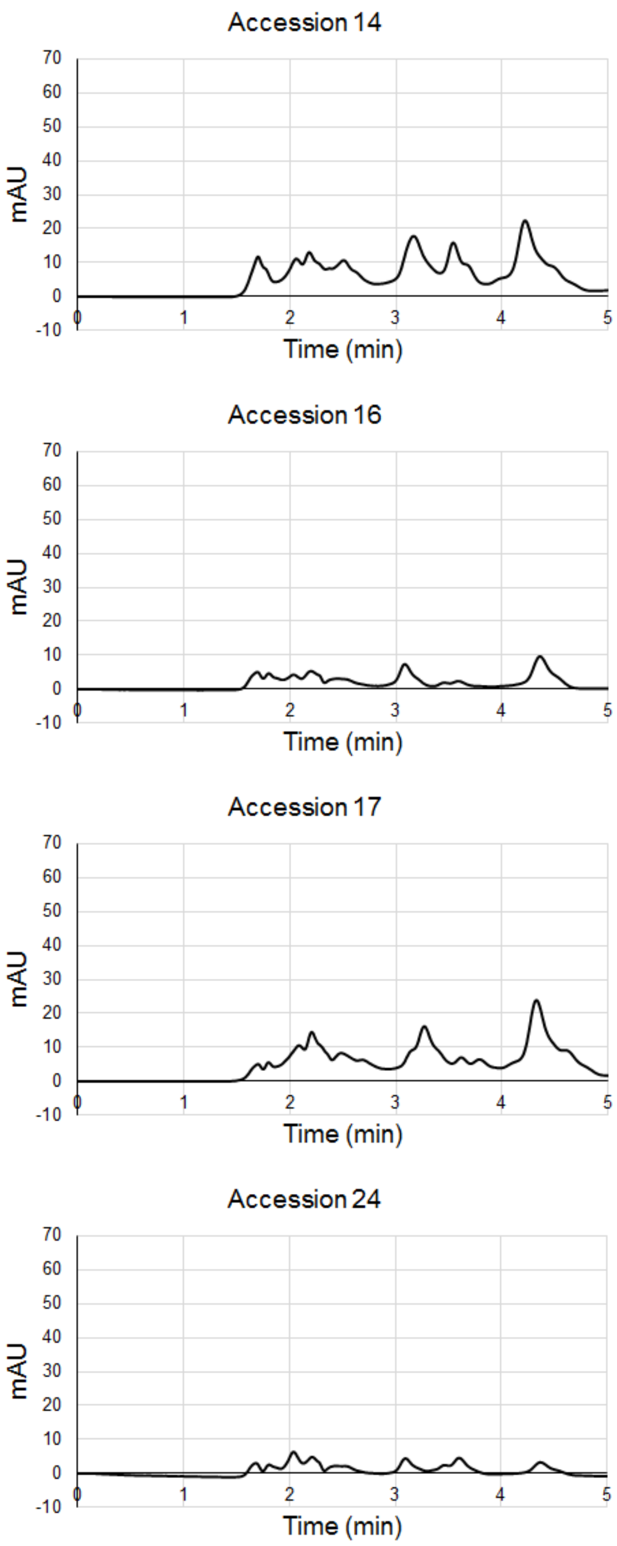

Supplementary Figure S2. HPLC analysis of ascorbate in standard, buffer and sample extracts. The retention time for ascorbate was 2.59 min. No ascorbate was found in any of the extracts.

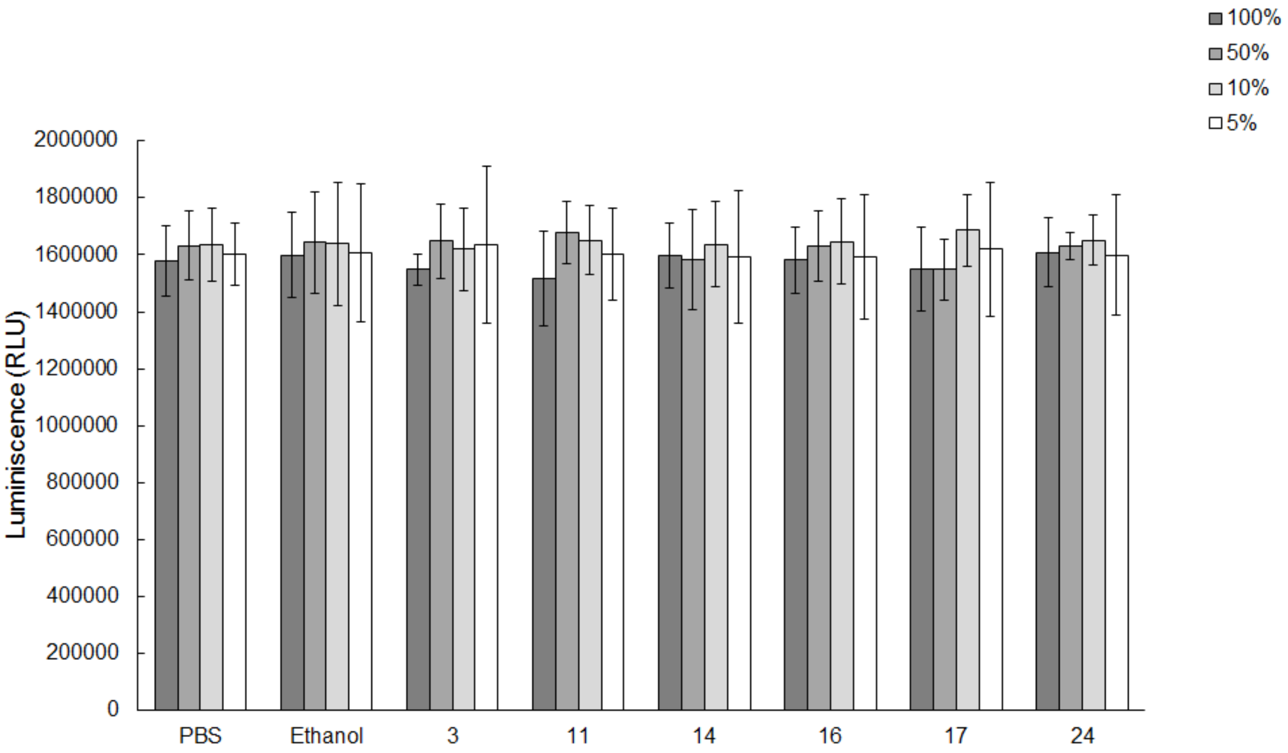

Supplementary Figure S3. There were no significant differences among the effects of different extracts on cell viability after incubation with the *Harpagophytum* extracts or control solutions (PBS and ethanol) (n=3). The diagram is illustrating mean values ± standard deviation.
